# How effective and cost-effective is water fluoridation for adults? Protocol for a 10-year retrospective cohort study

**DOI:** 10.1038/s41405-021-00062-9

**Published:** 2021-01-21

**Authors:** Deborah Moore, Thomas Allen, Stephen Birch, Martin Tickle, Tanya Walsh, Iain A. Pretty

**Affiliations:** 1 Dental Public Health, Division of Dentistry, Dental Health Unit, The University of Manchester, Lloyd Street North Manchester Science Park, Manchester, M15 6SE UK; 2 Director, Centre for the Business and Economics of Health, University of Queensland, St Lucia, Queensland, 4072 UK; 3 Health Economics, Manchester Centre for Health Economics, The University of Manchester, Jean McFarlane Building Oxford Road, Manchester, M13 9PL UK; 4 Dental Public Health & Primary Care, Division of Dentistry, The University of Manchester, Coupland 3, Manchester, M13 9PL UK; 5 Healthcare Evaluation, Division of Dentistry, The University of Manchester, Coupland 3, Manchester, M139PL UK; 6 Public Health Dentistry, Division of Dentistry, Dental Health Unit, The University of Manchester, Lloyd Street North Manchester Science Park, Manchester, M15 6SE UK

**Keywords:** Fluoridation, Dental treatments

## Abstract

**Background:**

Tooth decay can cause pain, sleepless nights and loss of productive workdays. Fluoridation of drinking water was identified in the 1940s as a cost-effective method of prevention. In the mid-1970s, fluoride toothpastes became widely available. Since then, in high-income countries the prevalence of tooth decay in children has reduced whilst natural tooth retention in older age groups has increased. Most water fluoridation research was carried out before these dramatic changes in fluoride availability and oral health. Furthermore, there is a paucity of evidence in adults. The aim of this study is to assess the clinical and cost-effectiveness of water fluoridation in preventing invasive dental treatment in adults and adolescents aged over 12.

**Methods/design:**

Retrospective cohort study using 10 years of routinely available dental treatment data. Individuals exposed to water fluoridation will be identified by sampled water fluoride concentration linked to place of residence. Outcomes will be based on the number of invasive dental treatments received per participant (fillings, extractions, root canal treatments). A generalised linear model with clustering by local authority area will be used for analysis. The model will include area level propensity scores and individual-level covariates. The economic evaluation will focus on (1) cost-effectiveness as assessed by the water fluoridation mean cost per invasive treatment avoided and (2) a return on investment from the public sector perspective, capturing the change in cost of dental service utilisation resulting from investment in water fluoridation.

**Discussions:**

There is a well-recognised need for contemporary evidence regarding the effectiveness and cost-effectiveness of water fluoridation, particularly for adults. The absence of such evidence for all age groups may lead to an underestimation of the potential benefits of a population-wide, rather than targeted, fluoride delivery programme. This study will utilise a pragmatic design to address the information needs of policy makers in a timely manner.

## Background

### Dental caries and fluoride

Dental caries, or tooth decay, is the leading global cause of disease, affecting 35% of the population.^1^ It a major public health problem with significant costs for both the individual and society. It can cause pain, sleepless nights, sepsis, overuse of antibiotics, embarrassment and the loss of productive workdays.^2^ It’s treatment is also very costly; across the 28 European Union countries, dental care costs are higher than those for Alzheimer’s disease, cancer, and stroke, with only diabetes and cardiovascular disease costing more.^3^ Despite the consistently high prevalence of dental caries globally, in high-income countries, prevalence in children has declined substantially over the past 40 years.^4,5^ This major success has been attributed to the increased use of fluorides for prevention, particularly in toothpastes.^6^

The caries-preventive effect of fluoride was first discovered in the first half of the 20th century, when a series of US studies reported that drinking water containing 1.0–1.2 mg of fluoride per litre was associated with a 50% lower caries prevalence.^7–9^ Decay occurs when dietary sugars are metabolised by intraoral bacteria to create acid waste products which can dissolve the mineral component of tooth enamel.^10^ At the earliest stage, when defects are still microscopic, fluoride promotes replacement of the lost mineral, helping to reverse the decay process.^11,12^ Fluoride was first added to public water supplies in the 1950s and became widely available in toothpastes from the mid-1970s.^6^ It has since been added to mouthwashes and professionally applied gels and varnishes.^13^ Improvements to oral health in the post-fluoride era have been dramatic: In 1973 the UK child dental health survey found that 97% of 15 year olds had experienced decay, compared to 42% in 2013.^5^

More than 70% of public water supplies in the US are now fluoridated, as are 89% in Australia.^14,15^ Within the UK, only England has implemented water fluoridation and since 1995 coverage has remained at around 10% of the population.^16^ Decisions on water fluoridation currently rest with Local Authorities and several areas of England are considering investing in water fluoridation to improve the dental health of their populations.^17–19^ Current estimates of cost-effectiveness suggest that after 10 years, every £1 spent on water fluoridation will lead to a £21.98 return, with savings due to reduced dental treatment costs and societal impacts of poor dental health such as absenteeism.^20^ However, as outlined below, the majority of the evidence upon which this estimate is based is more than 40 years old and does not take-into-account the dramatic improvements in dental health that have been experienced within that time frame as a result of the increased availability of topical fluoride products.

### Existing literature

There have been three landmark reviews of the evidence underpinning water fluoridation in the UK. In 2000 the York Centre for Research and Dissemination, commissioned by the Department of Health, undertook the first systematic review.^21^ The York review found the majority of included studies demonstrated a beneficial effect of water fluoridation for preventing caries in children, but the quality of the evidence was low. In 2002, the Department of Health requested the Medical Research Council (MRC) to make recommendations for future research priorities.^22^ These included:

- The impact of water fluoridation on caries reduction in children against a background of widespread topical fluoride use (for example, in toothpastes, gels and varnishes).
- Economic impacts and the effects of fluoridation on health and wellbeing beyond the usual measures of decayed, missing and filled teeth.
- The effect of fluoridation on social disparities in dental caries.
- Effects of fluoridation on the dental health of adults.

In 2015 Cochrane Oral Health carried out a systematic review of water fluoridation.^23^ The included studies were assessed as being at high-risk of bias and most had been carried out prior to the widespread introduction of fluoride toothpastes in the mid-1970s. When analysed in a separate sub-group analysis, the post-1975 studies no longer demonstrated a protective effect. Furthermore, no studies which included adults met the review’s inclusion criteria. The reviewers concluded that whilst there was historical evidence of a caries-protective effect, the lack of contemporary evidence made it difficult to determine if water fluoridation remains effective.

In response to the urgent need for more recent evidence on the effects of water fluoridation in children, the UK National Institute of Health Research (NIHR) funded ‘CATFISH’ study is currently underway in Cumbria, UK. It is hoped that this 7-year prospective cohort study will address many of the research priorities first posed by the MRC almost 20 years ago.^24^ However, it will not address the effectiveness of water fluoridation for adults and no other such studies are in progress. Studies including adults are increasingly important due to changing disease patterns and demographics. Fluoride and increased access to restorative dentistry mean that most adults today can expect to retain some natural teeth for their whole lifetime.^25^ Whilst this is a positive outcome, there is increasing evidence that the majority of caries-free children do not remain so as adults.^26–28^ The highest prevalence and incidence of tooth decay in permanent teeth is now thought to occur in adolescents and older adults.^29–33^

Dry mouth is a common side effect of many long-term medications and wearing partial dentures, dementia and dependency on others for mouthcare, can all increase an older person’s risk of tooth decay.^32^ Older people are also more likely to have gingival recession, which exposes the more susceptible root surfaces to decay.^31^ After a lifetime of repair, teeth can become heavily filled and fragile. Restoring new cavities or replacing old crumbling fillings in such teeth can be technically demanding and hence, costlier. Difficulties with consent and co-operation due to advanced dementia or medical complexities can mean that routine dental care is no longer possible for some older people and management is limited to pain relief and control of sepsis.^34,35^ With the number of people aged over 65 projected to increase by 71% between 2010 and 2050 in developed countries,^36^ there is an urgent need to understand the effectiveness and cost-effectiveness of different caries-preventive strategies for adults.

### Challenges of studying water fluoridation in adults

There are several reasons why the recent Cochrane systematic review did not identify any studies on the effect of water fluoridation on adult dental health.^23^ Firstly, the inclusion criteria specified prospective studies with a concurrent control, comparing two populations whose fluoridation status was the same at baseline and subsequently changed. The opportunity for such studies was greatest during the 1970s and 1980s, when the majority of new schemes were introduced. At that time the strategic focus was caries-prevention in children, because the norm was almost universal experience of disease by adolescence.^37^ With changing disease patterns there is increasing awareness of the need to prevent caries in adults,^38,39^ but less opportunity to study the effects of new water fluoridation schemes because coverage has either reached near-maximum levels (US and Australia), or stalled (UK). Several authors have critiqued the Cochrane review’s inclusion criteria as being unfeasibly stringent.^40,41^ They argue that the majority of contemporary evaluations relate to surveillance of existing schemes, therefore, the most feasible and realistic study designs are well-controlled cross-sectional or cohort studies.^40,41^

Secondly, there are logistical difficulties in recruiting large numbers of adults and following them over several years, as is required in studies of caries development. Groups of children can be recruited, examined and followed-up relatively efficiently with the support of schools and nurseries. Even with this help, the costs of repeated clinical examinations by trained and calibrated dentists are high. For example, the ongoing CATFISH study in children is funded at £1.6 million.^42^ A comparable community-based setting for adults does not exist, so recruitment and follow-up would either be prohibitively time and resource intensive, or would risk significant loss to follow-up.^43^

Thirdly, measuring exposure to water fluoridation is more challenging in adults than in children. Assigning water fluoridation exposure status to individuals requires some knowledge of where they have lived within specific timeframes. Obtaining an accurate residential history is more difficult in adults than children, due to the longer recall periods involved. Recent studies in adults that utilised participant recall have suffered from missing data and large numbers of exclusions.^41,44–49^

Finally, measuring caries progression in older adults is also particularly challenging. Progression is usually recorded by counting the number of teeth, or tooth surfaces, affected by decay and noting any increase in the count over time. However, when new decay occurs on a previously affected tooth or tooth surface, the count does not increase. This ‘ceiling’ effect is a particular problem in older adults when many teeth and surfaces have already been affected by decay. A recent study on the effects of water fluoridation in Australian adults did not  demonstrate any benefit in the age groups over 45, which the authors attributed to this measurement ceiling.^46^ Studies in the UK and Australia have also shown that it is the number of extracted teeth, rather than the count of decayed teeth or surfaces, that is the best measure of oral health disparities in older adults.^50,51^

## Methods/design

The study design is a retrospective cohort study using anonymised, routinely collected electronic billing records of individuals who received NHS dental care in England within the last 10 years (T—10 years, with exact date ‘T’ depending on the day of data download). We will utilise these records to explore differences in the number and types of dental treatments provided for patients receiving fluoridated or non-fluoridated water.

Variation in water fluoridation coverage across England means that individuals will have differential levels of exposure, depending on where they have lived. As described in the MRC guidance on ‘natural experiments’, this type of variation offers opportunities for evaluating public health interventions, where random allocation of individuals or clusters to intervention groups is not possible.^52^

**Fig. 1:**
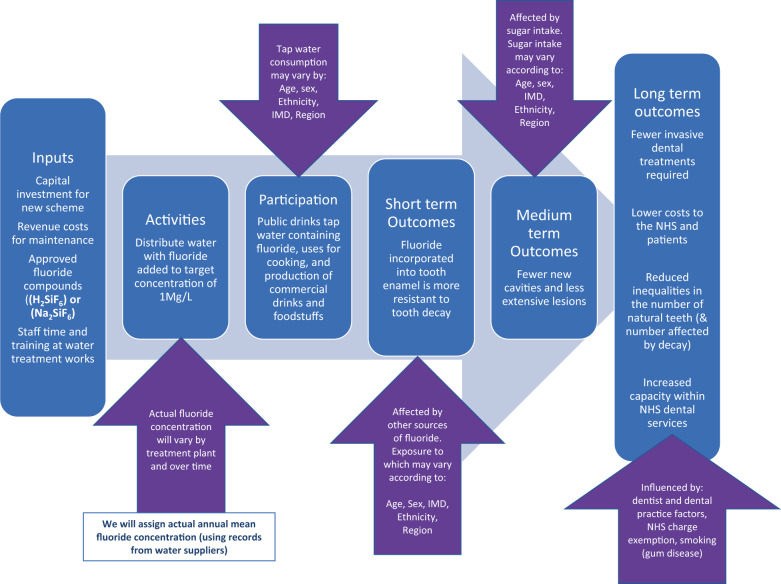
Logic model for water fluoridation. A ‘theory of change’ illustrating how programme inputs create public health outcomes (columns) and the factors that may influence this process (arrows).

A logic model is presented in Fig. 1 illustrating the steps involved in successful delivery of water fluoridation and the factors which may influence programme delivery and outcomes.

### Rationale for the proposed study design

Conducting research on the effects of water fluoridation in adults involves significant methodological challenges. This has resulted in a paucity of evidence and recommendations for research that have not been addressed in 20 years.^22,53^ The present study has been designed to contribute to the evidence base, in a pragmatic and cost-efficient way, using routinely collected NHS dental treatment data.

Using routine data has several advantages over traditional designs using clinical epidemiological surveys:

- The costs of using existing data are much lower than a clinical examination study that would require significant input in terms of recruitment, clinical facilities and clinician time.
- The data has already been collected, avoiding the problems of loss to follow-up and long waiting times for decision makers.
- To address questions on cost-effectiveness requires real-world information on treatment decisions and use of resources, which are available in NHS dental datasets.
- Using dental treatment as an outcome allows us to meet the MRC recommendation that researchers study ‘the effects of fluoridation on health and wellbeing beyond the usual measures of decayed, missing and filled teeth [DMFT]’.^22^ Avoiding unpleasant and costly dental treatment was seen as particularly important by the dental patients and members of the public that we spoke to during the design stage of this study.
- Using dental treatment data allows for consideration of the lifetime consequences of recurrent decay, including the ‘repeat restoration cycle’.^54^ This will avoid the measurement ceiling observed in older people when using the traditional count of the number of decayed, missing and filled teeth (DMFT).

### Description of the data source

The National Health Service (NHS) offers state-subsidised dental care to all adults and fully-funded dental care to specific groups, including children under 18.^55^ Using published literature on dental attendance and use of NHS dentistry, we estimate that ~79% of the English adult population will have attended an NHS dental practice between 2010 and 2020.^56–58^ The NHS Business Services Authority (BSA) are an ‘arms-length’ body of the Department for Health and Social Care, responsible for processing payment claims made by dentists for the NHS treatment they provide.^59^ This study will utilise the NHS BSA Dental Claims dataset for England.^60^ The dental claims data is stored for a period of 10 years, and the NHS BSA are able to link together discreet courses of treatment provided for one individual by matching their patient identifier (surname, initial, gender and date of birth) to their unique 10-digit NHS number. The number and types of dental treatment received can be identified even when provided by different NHS dentists in different areas of the country.

The record also contains the unique ‘NHS performer number’ of the dentist who carried out the treatment. Using this number, it is possible for the NHS BSA to add the age, gender, place of qualification and year of qualification of the treating dentist. Information about the dental practice, such as the size and type of contracting arrangement and the financial value of the contract is also available. With regards to individual patients, the dataset includes their home address at each course of treatment, their age, gender and ethnicity. Importantly, it also includes an individual-level measure of socio-economic status, in the form of the NHS charge exemption category. The reason for any fee exemption or remission must be recorded at every course of treatment. Reasons include being in receipt of welfare payments due to low income, being out-of-work, or long-term ill health; as well as being aged 18 in full time education, being in prison, being pregnant or having had a baby in the previous 12 months.^55^

### Constraints of using routinely collected data

There are inevitably some constraints when using an existing dataset that was not designed for research. The NHS BSA dataset is structured around courses of dental treatments. To create a longitudinal record for an individual requires the patient’s unique NHS number. Initial scoping by the NHS BSA suggests that around 50% of the records they hold can be linked in this way, which will limit the size of the cohort available for analysis. We will investigate any potential impact on generalisability by asking the BSA to compare the demographic characteristics of those records with and without an NHS number. We expect that the recorded dental treatment will be an accurate reflection of NHS care, because the claim form is signed by the patient and claims are randomly checked for fraud by the BSA. However, any treatments provided privately are not recorded. This may occur if, for example, the dentist recommends a metal crown or filling in a molar tooth, but the patient requests a more aesthetic, tooth coloured option. Since 2017 the number of ‘decayed, missing and filled teeth (DMFT)’ has also been recorded. However, this relies on the dentist completing an accurate dental charting and keeping it up to date, which audits have shown may not always happen.^61,62^

The NHS BSA dataset does not include diagnosis, so some treatments will have been provided for non-caries reasons, such as periodontal (gum) disease, or injury. This will not present a problem in detecting fewer treatments due to caries, because a relative difference between groups will be evident. This is true unless there is another, known or unknown reason, why non-caries treatments would vary systematically by intervention group. It is possible that dentists in fluoridated areas may perform a greater number of discretionary treatments such as replacing old or worn fillings, because of less time pressure and/or a desire to maintain income when practising in low-caries populations. More frequent replacement of old fillings would increase the likelihood of a null finding if this occurs at sufficient volume. If we do indeed fail to detect a difference between groups, systematic differences in the treatment behaviour of dentists in response to lower caries levels would need to be considered as a possible explanation.

It is well-established that dentists have different thresholds for surgical intervention when faced with the exact same scenario of caries progression.^63^ Random inter-operator variation would not introduce measurement bias as it would be equally distributed between intervention and control groups. However, any variation which is associated with the patient’s likelihood of being in the fluoridated or non-fluoridated group could do so. Factors which are known to be related to the intervention threshold of dentists include age, dental school, access to continued professional development, size of practice, gender and remuneration system.^64,65^ Some of these factors could feasibly show geographic clustering, for example, in cities or closer to dental schools. Because there are relatively few fluoridated areas in England, it could occur by chance that clusters of such dentist and dental practice factors are unequally distributed across intervention groups. We will account for any such imbalances during the analysis stage.

The dataset does not contain any information about patient behaviours which are strongly related to oral health, such as toothbrushing, sugar intake and smoking. Potential confounding due to non-random variation in these behaviours will be addressed by taking into account their underlying social determinants, such as age, gender, area-based deprivation (Index of Multiple Deprivation), ethnicity and income-related exemptions from NHS dental charges.^66,67^ Most of these fields will be well completed as they are essential requirements on every form. The exception is ethnicity, which the patient is asked to complete. Depending on the extent of the missing data for this field, we may need to account for ethnicity using area-based measures and/or via a sensitivity analysis restricted to complete records. The wider, societal costs associated with oral health problems and their treatment, such as absenteeism and presenteeism with relation to work or school, cannot be measured from the routine data available in this study. However, it is not expected that the relationship between treatments and these costs will differ between fluoridated and non-fluoridated regions.

#### Aim

To pragmatically assess the clinical and cost-effectiveness of water fluoridation for preventing dental treatment and improving oral health in a contemporary population of adults, using a natural experiment design.

#### Primary objective

- To compare the effect of 10-year exposure to fluoridated water with no exposure, on the number of invasive dental treatments, including restorations (fillings), endodontics or extractions, received by adults attending NHS dental practices.

#### Secondary objectives

- To evaluate the cost-effectiveness of water fluoridation in reducing the amount of invasive dental treatment in an adult population with 10-year exposure to fluoridated water when compared to a population with no exposure, taking a public sector perspective.
- To estimate the return on investment from a public sector perspective in terms of the change in the cost of providing dental treatments generated from an investment in water fluoridation.
- To compare the impact of 10-year exposure to water fluoridation with no exposure on the oral health (number of remaining natural teeth and decay experience [DMFT]) of adults attending NHS dental practices.
- To measure the impact of 10-year exposure to water fluoridation on social inequalities in oral health in adults attending NHS dentists.

#### Participants

Adults and adolescents aged over 12 years, attending NHS dental practices in England in the last 10 years (T—10 years). Adolescents aged over 12 were included as this is the age at which the permanent, adult teeth are usually present in the mouth (except for third molars, or ‘wisdom teeth’).^68^

### Inclusion criteria

1. Dental records that can be assigned to a unique individual using the combination of NHS BSA Identifier (initial, surname, gender, D.O.B) and NHS number.

### Exclusion criteria

1. Individuals will be excluded from further analysis if they do not have at least two episodes of dental attendance, within the 10-year observation period (T—10 years).

### Exposure definition

Determining an individual’s level of exposure to water fluoridation represents a major challenge for conducting research on its effects (Fig. 1). The implementation of the UK target dose of 1 Mg F/L is inconsistent both over time and at different water treatment plants.^16,69^ Equipment failures, unexpected weather events and difficulty in obtaining the correct fluoridation chemicals mean there are some water fluoridation plants which have had periods of inactivity or have been producing water which is sub-optimally fluoridated for a number of years.^16^ A recent study found that over a period of 18–35 years, the achieved mean water fluoride concentrations of eight water treatment plants in England varied from 0.53 Mg F/L (SD 0.47) to 0.93 Mg F/L (SD 0.22) Mg F/L, with a range of 0.00 Mg F/L to 1.26 Mg F/L.^69^

We propose to quantify exposure to water fluoridation for individuals, for the defined 10-year exposure and observation period (T—10 years). It is accepted that we do not know where the participants have lived prior to the 10-year period or what their exposure to water fluoridation has been historically. In view of the main method of action of fluoride now being understood to be topical^70^ and the fact that caries has been estimated to progresses at a rate of around 0.8–1.2 new surfaces per year in adults,^28,33,71^ we would expect to see some difference in the number of dental treatments received due to new caries over a period of 10 years, even for those who moved into the fluoridated region at the start of the observation period. The advantage of this pragmatic approach is that we do not need to restrict our sample to participants who have lived in fluoridated or non-fluoridated regions since childhood. Such a criterion would make any study extremely difficult to recruit to, resulting in a small sample size^47^ and would also result in a skewed sample that is unlikely to be generalisable to the wider population.

Annual water fluoride concentrations must be recorded by water companies as part of routine water quality monitoring.^72^ We will obtain this information and compile a record of annual water fluoride concentrations (Mg F/L) for England that can be linked to patient place of residence. This will involve some approximation using Geographic Information System mapping, as water geographies are not aligned with standard UK geographies such as postcode areas or local authority boundaries. We will then create a 10-year exposure profile (Mg F/L) for each individual, based on how many years they lived within each region and what the annual water fluoride concentration was during that time period. We will then summarise the average 10-year water fluoridation exposure for each individual, using either mean and standard deviation, or median and IQR (depending on distribution).

For the main analysis, we will group individual participants according to their personal residential water fluoride concentration over the 10-year period (T—10 years):

- *Exposed*: Individuals who have lived in lower super output areas (LSOAs) with an average fluoride concentration of ≥0.7 mg f/l. This is estimated to be ~10% of English population.^16^
- *Un-exposed*: Individuals who have lived in LSOAs with an average fluoride concentration of <0.7 mg f/l. This is estimated to be ~90% of English population).^16^

This approach to exposure classification will not differentiate between fluoride that is in the water as a result of geology (naturally fluoridated), or as a result of a public health programme (artificially fluoridated). However, variability in implementation of water fluoridation programmes and the effect of achieved fluoride concentration is important and will be considered in ancillary analyses.

### Comparator group

A key consideration in the design of this study is the selection of an appropriate comparator group, to minimise bias and to strengthen causal inference. Decisions regarding the implementation of water fluoridation are currently made by local authorities. Important factors in these decisions are population oral health, population size, the complexity of the local water system and local government politics.^17^ Therefore, the likelihood of an individual receiving water fluoridation is related to such place-based factors.^73^ Place-based factors may also influence the likelihood of an individual receiving different types of dental treatment. Examples include; dentist: population ratio, population oral health, proximity to a dental school, and availability of secondary care referral services. In order to ensure these place-based factors are taken into account during the analysis, we propose to compare exposed and unexposed individuals from local authority areas that are most similar to each other on a range of place-based characteristics.

Selection of the characteristics for matching of local authority areas will be undertaken in partnership with key stakeholders including clinicians, public health specialists, statisticians and policy makers. Similarity of local authorities based on these selected characteristics will be formally assessed using propensity scores.^74,75^ Following the creation of balanced propensity scores, local authority areas will be matched using nearest neighbour matching or ‘greedy’ matching using the ‘MatchIt’ package in R.^75,76^ Matched sets of local authority areas will be formed using one to many matching (with a ratio of no more than 1:5 of intervention local authority units to controls), based on similar values of the estimated propensity score.

A descriptive analysis will be undertaken to determine whether balance at the local authority level has been achieved. Average standardised absolute mean differences will be calculated as a global measure of successful matching. This approach is preferable to statistical significance tests which can, in large datasets, be overly sensitive to observed differences. Expert knowledge of local areas will also be used to qualitatively assess success.

### Estimated sample size

We estimate that that after exclusions and matching of local authority areas, the final sample size of the study will be approximately six million individuals.

#### Primary outcome measure

- Number of invasive dental treatments (restorations, endodontics and extractions) received by adults attending NHS dental practices over 10 years of observation (T—10 years).

#### Secondary outcome measures

- Mean cost per episode of invasive dental treatment avoided.
- Total number of natural remaining teeth (routinely recorded in NHS BSA dataset from 2017).
- Total number of teeth affected by decay (DMFT) (routinely recorded from 2017).

### Analysis of clinical outcomes

A generalised linear model with clustering by local authority area will be used to analyse the primary outcome of number of invasive dental treatments received during the period of observation. This model will include the area level propensity score and individual-level covariates. Given the large number of observations, clinical importance of the magnitude of the treatment effect will be preferred over statistical significance. Thresholds for minimally important differences will be defined a priori in partnership with key stakeholders including decision makers, public health professionals, patients, clinicians and the public.

### Economic evaluation

The economic evaluation of water fluoridation will focus on an assessment of cost-effectiveness and calculating the return on investment.

Cost-effectiveness will be based on the primary study outcome, assessed as the mean cost per episode of invasive dental treatments avoided, from a public sector perspective, by estimating the incremental cost-effectiveness ratio (ICER). The ICER is measured by the difference in water fluoridation costs between fluoridated and non-fluoridated regions (incremental costs), divided by the difference in the number of invasive dental treatments between fluoridated or non-fluoridated regions (incremental effects). Reductions in dental service costs are not factored into the incremental costs of fluoridation (numerator in the ICER) because the reduction in treatment episodes is used as the measure of effects (denominator in the ICER). Deduction of the costs of reduced dental treatments from the costs of water fluoridation would involve ‘double counting’ this change in treatment episodes and these effects on costs are instead considered in an estimation of the financial return on investment. Sensitivity analysis will determine if cost-effectiveness is impacted by characteristics of the population or water fluoridation scheme.

To provide valuable information to policy makers, we will calculate the public sector financial return on investment in water fluoridation. The investments will be captured by the costs of providing water fluoridation, while the returns will be captured by changes in NHS costs relating to reductions in dental service utilisation.

#### **Cost of dental treatments**

In order to assess the financial return on investment associated with fewer dental treatments in fluoridated areas we will measure the following:

1. *NHS* *costs***:** Contracts to provide NHS dentistry in England consist of an agreed annual financial payment, combined with a defined level of expected annual activity. Activity is measured in Units of Dental Activity (UDAs), which are accrued based on the complexity of care provided within each course of treatment. The financial value of UDAs varies across the country, based on historical arrangements, but the average value in 2018/9 was £27.04.^77^
  A Band 1 course of treatment (examination, prevention, radiographs) attracts 1 UDA (for which the NHS pays £27.04). A Band 2 course of treatment (restoration, endodontics, extractions) attracts 3 UDAs (for which the NHS pays £81.12). A Band 3 course of treatment (crowns, dentures) attracts 12 UDAs (for which the NHS pays £324.48).
2. *Patient costs***:** Where patients are not exempt from NHS charges, they pay a portion of the above total NHS costs. The proportion of the full NHS cost that is paid by patients has increased in recent years. In 2018/9 the patient charges for each band is as follows: Band 1 (£22.70), Band 1 Urgent (£22.70), Band 2 (£62.10), and Band 3 (£269.30).^78^ Patient costs will be allocated using the true costs for the year in question. Patient costs relating to the time and travel required for dental treatments cannot be measured using data available for this study.
3. *Cost per item of treatment:* Payment bands will also be disaggregated to extract a more precise cost of the treatment provided within each band. This will involve assigning a unit cost per item of treatment. NHS dental costs are still assigned in this way in Scotland based on the estimated mean time taken to provide different items of service, so we will utilise Scottish dental treatment costs data as a more ‘resource-based’ approach to costing.

Patient costs will be deducted from NHS costs to reflect that patient charges are recovered by the NHS and are a source of income. Each costing approach will be applied to the patient level data for patients living in fluoridated and non-fluoridated regions.

The costs of dental treatments and the number of treatments avoided will be discounted at 1.5% which is the UK National Institute for Health and Care Excellence (NICE) recommended discount rate for public health interventions. Sensitivity analysis will apply the 3.5% discount rate common for health care interventions.

#### Cost of fluoridation

Costs of water fluoridation involve capital expenditure for equipment, and ongoing revenue costs, which include; maintenance, training of operators, the time taken by water company staff, and the fluoride chemical supply. Public Health England, on behalf of the Secretary of State, fund capital costs.^17^ Revenue costs are paid by Public Health England and subsequently recharged to Local Authorities. Capital costs will also need to consider the estimated lifetime of the plant and any major refurbishments required. Capital and revenue costs of fluoridation will be obtained by liaising with Public Health England and the appropriate water companies. Fluoridation costs will be allocated appropriately to the whole population in each fluoridated region to calculate the per capita cost. As costs do not vary by patient characteristics, the per capita cost will be applied to our patient population. We will determine the degree to which the cost of water fluoridation is driven by fixed costs and variable cost, the latter varying with the size of the population served.

## Discussion

Several areas of England are currently considering investing in water fluoridation to improve the dental health of their populations.^18,19^ Water fluoridation proposals are controversial, with some groups and individuals vehemently opposed.^79^ In 2007, the Nuffield Council on Bio-ethics developed an ethical framework specifically for public health, where they considered the case of water fluoridation.^80^ The guidance determined that there is no ethical prohibition against adding beneficial substances to the water supply to improve health and reduce health inequalities, even when some individuals oppose it. Rather, the decision should be made through local democratic processes and should consider the balance of risks and benefits expected, the potential for alternative interventions which do not compromise autonomy to the same extent and the role of consent if there are expected harms. Legislation states that any proposal to introduce water fluoridation in the U.K. must include a 3-month public consultation period where these issues can be explored.

Whilst there are many spurious and scientifically implausible claims of harm from water fluoridation,^79^ there is one well-recognised unwanted effect. Dental fluorosis is an increased porosity of tooth enamel which may be observed as brown or white flecks on the permanent teeth. It occurs as a result of fluoride ingestion during the time that the tooth enamel is forming, between birth and 8 years of age. A recent study in England estimated the prevalence of ‘aesthetically objectionable’ fluorosis to be around 10% in fluoridated cities, compared to 2% in non-fluoridated cities.^81^ There is no lower ‘threshold’ dose for dental fluorosis. Instead, prevalence increases linearly with every increase in dose above 0.01 mgF/kg of bodyweight per day.^82^ Prevalence is thought to have increased in recent years, due to inadvertent ingestion of topical fluorides such as toothpastes and varnishes.^46,81,83,84^ Some countries, including Ireland and the U.S., have reduced the target dose for water fluoridation programmes as a result.^85–87^

It has been suggested that a targeted approach, using topically applied fluoride products, may be a more feasible and acceptable strategy for caries-prevention.^79,88,89^ Such an approach offers the opportunity for individuals to consent or dissent. For those who do ingest the topical fluoride products inadvertently, if they have been targeted as ‘high-risk’ for caries, the benefit: risk profile would be more favourable than in a population-wide approach. For a targeted, or high-risk approach to be efficient, the disease must have a low enough prevalence to make the extra effort involved with identifying those at risk cost-effective.^90^ The most recent children’s dental health survey found that 44% of 15 year olds in England had experienced decay in their permanent teeth, which is higher than the 30% above which a targeted approach is no longer practical.^90,91^ Recent estimates from national surveys across western Europe demonstrate that by the age of 35–44, the prevalence of decay experience is at least 92%.^92^ With sufficient time, caries remains almost universal and the difficulties of accurately predicting which individuals or communities are at highest risk are well recognised.^93,94^ Undoubtedly, population-wide interventions to prevent caries remain essential if the greatest burden of disease is to be avoided.

It is important to note that the most fundamental component of any population-wide caries-prevention strategy is sugar reduction through the use of upstream policy levers.^2^ Reducing the underlying cause of disease through the restoration of normality, rather than adding a protective factor, is a more radical approach, and does not come with the risk of unwanted effects such as dental fluorosis.^95^ However, with any highly prevalent chronic disease, the social and biological causal pathways involved are complex and action at multiple levels is required.^96^ Fluoride is highly effective at preventing caries and its use over the last 50 years has transformed dental health. Whether it should be applied in targeted programmes or at the population level is the key question for local communities. Within these debates, it is imperative that the benefits for the whole population, including the health care system, can be articulated. We recognise our pragmatic study design comes with some limitations in terms of causal attribution and our results will need to be triangulated alongside the range of contemporary evidence for water fluoridation. At the same time, real-world data offers meaningful evidence of impact and the ability to capture the most important benefit of a population-wide approach.
